# Pseudo-Reference-Based Assembly of Vertebrate Transcriptomes

**DOI:** 10.3390/genes7030010

**Published:** 2016-02-24

**Authors:** Kyoungwoo Nam, Heesu Jeong, Jin-Wu Nam

**Affiliations:** 1 Department of Life Science, College of Natural Sciences, Hanyang University, Seoul 04763, Korea; nkw0228@hanyang.ac.kr; 2 College of Liberal Studies, Seoul National University, Seoul 08826, Korea; hs0517@snu.ac.kr; 3 Research Institute for Natural Sciences, Hanyang University, Seoul 04763, Korea

**Keywords:** transcriptome assembly, RNA-seq, pseudo-reference

## Abstract

High-throughput RNA sequencing (RNA-seq) provides a comprehensive picture of the transcriptome, including the identity, structure, quantity, and variability of expressed transcripts in cells, through the assembly of sequenced short RNA-seq reads. Although the reference-based approach guarantees the high quality of the resulting transcriptome, this approach is only applicable when the relevant reference genome is present. Here, we developed a pseudo-reference-based assembly (PRA) that reconstructs a transcriptome based on a linear regression function of the optimized mapping parameters and genetic distances of the closest species. Using the linear model, we reconstructed transcriptomes of four different aves, the white leg horn, turkey, duck, and zebra finch, with the *Gallus gallus* genome as a pseudo-reference, and of three primates, the chimpanzee, gorilla, and macaque, with the human genome as a pseudo-reference. The resulting transcriptomes show that the PRAs outperformed the *de novo* approach for species with within about 10% mutation rate among orthologous transcriptomes, enough to cover distantly related species as far as chicken and duck. Taken together, we suggest that the PRA method can be used as a tool for reconstructing transcriptome maps of vertebrates whose genomes have not yet been sequenced.

## 1. Introduction

High-throughput RNA sequencing (RNA-seq) provides unbiased, genome-wide sequencing of RNA, allowing comprehensive understanding of an entire set of cellular RNAs (called a transcriptome) from a single cell to the individual level [1,2,3,4,5]. Taking advantage of the RNA-seq technique [6,7,8,9,10], recent large-scale studies have reported pervasive transcription signals on genomes in diverse cell-types [11,12] and unprecedented complex transcription structures in genomes [12,13,14,15,16,17,18,19,20,21]. A comprehensive map of the transcriptome can be acquired through the computational assembly of RNA-seq reads [22].

According to the availability of the relevant reference genome, two alternative computational approaches for transcriptome assembly can be applied, the *de novo* and reference-based approaches. The *de novo* transcriptome approach assembles transcripts by connecting short reads without a reference genome [22,23,24,25,26,27,28]. In contrast, the reference-based approach assembles transcripts by mapping the reads to a reference genome [29,30,31,32,33,34]. Compared to the *de novo* approach, the reference-based approach uses fewer computational resources and less time, and generally displays greater sensitivity and specificity in reconstructing previously annotated genes. Better performance is mainly attributed to the fact that the reference genome provides guidance for assembly, and is better for detecting low-copy transcripts and complex isoforms and for avoiding artifacts of transcripts such as chimeric transcripts [2,35].

Accurate assembly of RNA-seq reads helps to construct complex transcriptional and post-transcriptional events, such as convergent and divergent overlaps of transcripts, sense overlap of transcripts, alternative promoters, alternative splicing, alternative untranslated regions (UTR), and to discover novel transcripts [2,35]. The quality of the assembly depends on both the sequencing depth of RNA-seq and the expression level of genes [2]. In other words, the greater coverage of reads on a certain gene provides a greater sensitivity in reconstructing transcripts of the gene. However, a gene with low-copy transcripts is more likely to be covered by a low number of gene reads, resulting in either failing or wrong assembly. On the other hand, the quality of the transcriptome assembly is also affected by the uncertainty of genome sequences. In general, a misassembled genome often leads to erroneous transcriptome assembly. For instance, repeat elements, including simple repeats, short tandem repeats, and transposons, could allow the reads be mapped to the multi-loci of the genome, causing ambiguous transcriptome assembly. Thereby, the unique mapping rate of the short reads could be a pre-indicator for the quality of transcriptome assembly.

In spite of the high performance of the reference-based approach, it has not been used in species whose reference genomes are not sequenced. Alternatively, the genome of a closely-related species can be used as a reference [36]. Comparative analyses of genomes and transcriptomes have shown that a great number of genes are well conserved across vertebrates in transcription units, promoter usage, alternative splicing and polyadenylations, and gene synteny [37,38,39,40]. Particularly, humans share approximately 97% homologous genes with chimpanzees, greater than 96% with other primates, and about 95% with other mammals, including mice. Although such closely related genomes may be a good pseudo-reference for transcriptome assembly, the optimal mapping parameter that gives the highest unique mapping rate in the pseudo-reference has not been modeled with the genetic distances from the corresponding reference.

In this study, we propose a pseudo-reference-based assembly (PRA) that utilizes the genome of a closely related species based on a linear regression model of the optimal mapping parameters and genetic distance between closely related genomes. Here, we built a model with the genomes of nine closely related species including five aves and four primates. Given a pseudo-reference within a certain evolutionary distance, estimated by orthologous transcriptomes, we were able to estimate the optimal mapping parameter that provides the highest unique mapping rate. Our method provides high quality gene annotations and facilitates the discovery of novel genes in closely related species while lacking its own sequenced genome.

## 2. Materials and Methods

### 2.1. Dataset and Preprocessing

To perform the PRAs and to evaluate the resulting assemblies, we chose only species where the respective genome and RNA-seq data across species were both available. Accordingly, four aves (chicken, turkey, duck, and zebra finch) and a subspecies (WLH) were selected in aves, and four primate species (human, chimpanzee, gorilla, and macaque) were selected in primates (Supplementary Table S1). All eight genome sequences were downloaded from the Ensembl database [41] (Galgal4 for chicken and WLH; UMD2 for turkey; BGI_duck_1.0 for duck; taeGut3.2.4 for zebra finch; hg19 for human; CHIMP2.1.4 for chimpanzee; gorGor3.1 for gorilla; and MMUL_1 for macaque). All RNA-seq data for transcriptome assembly were downloaded from the NCBI gene expression omnibus (GEO) and short read archive (SRA) (GSM1419093 for WLH; GSM913024 for turkey; SRX255710 for duck; SRX493921 for zebra finch; SRX843148 for chimpanzee; GSM1064832 for gorilla; and SRX724878 for macaque). Before mapping the RNA-seq reads, we first evaluated the sequence quality of the reads using the FastQC (version 0.10.1), which checks whether the reads contain an adapter sequence. Based on the results, we trimmed the adapter sequence and nucleotides with low-quality using Sickle (version 1.2) [42] and Cutadapt (version 1.9.dev1) [43] with parameters of minimum base quality at 20 and minimum sequence length after trimming of 20 nt. After the trimming process, fragments without their paired fragments were removed as the *de novo* assembler programs only takes paired-end reads. For marker sequences for phylogenetic analysis, 16s ribosomal DNA and D-loop sequences of respective species were downloaded from NCBI [44] and one-to-one orthologous genes were downloaded from OMA [45,46].

### 2.2. Decision Flowchart for Pseudo-Reference-Based Assembly

**Figure 1:**
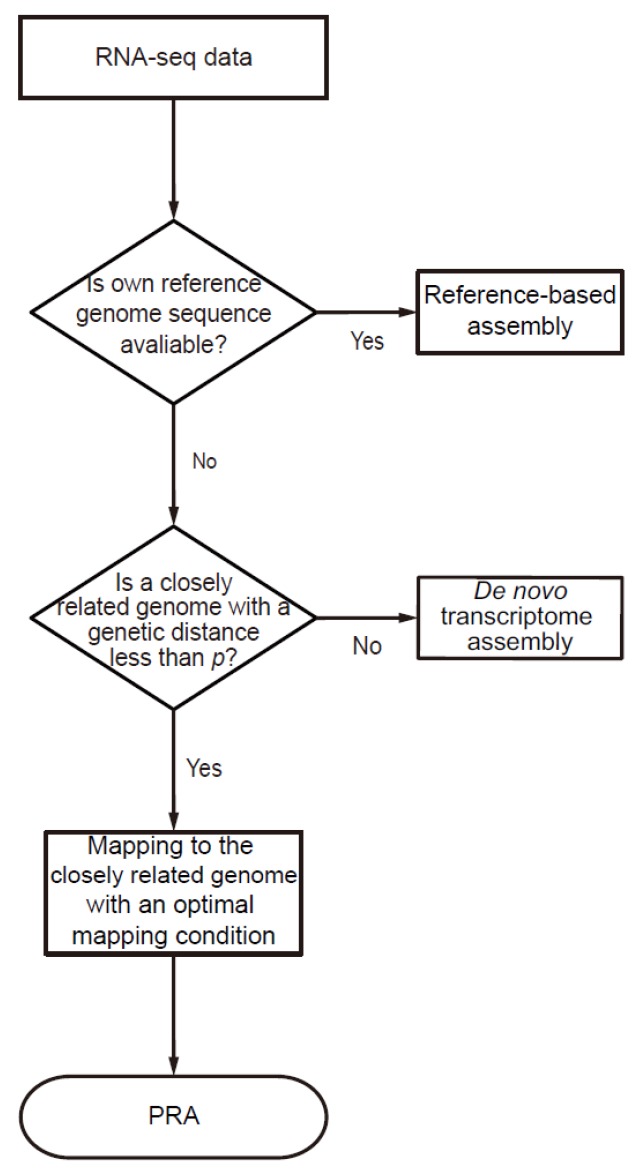
Decision flow system of transcriptome assembly (*de novo*, reference-based, and PRA). Given the RNA-seq from a certain species, if a reference is available, reference-based assembly is recommended. Otherwise, it asks whether the genome sequence of a closely related species is available within a certain genetic distance *p*. If available, the PRA is recommended. Otherwise, the *de novo* assembly is recommended.

To reconstruct the transcriptome in a species whose genome is not available, we developed a decision flow system for PRA with short RNA reads sequenced from the corresponding species (Figure 1). The decision flow system first checks the availability of the reference genome of the interested species. If available, the system recommends that the user pursue the reference-based assembly. Otherwise, the system next asks whether a closely related species is available within a genetic distance less than *p*. If not, the system recommends that the user pursue a *de novo* assembly. Otherwise, the pipeline estimates the optimal mapping parameter using three optimal mismatch rate models, trained from PRA experiments of seven vertebrates, where optimal mismatch rate is defined as the maximum allowed rate of mismatches in a read while in alignment, which maximizes the number of uniquely mapped reads (Supplementary Table S1). With an optimal mapping parameter, the pipeline performs the PRA with the best unique mapping rate. The criterion *p* is the genetic distance where the PRA outperformed the *de novo* assembly in terms of the reconstruction rate, which is described in the later section.

### 2.3. Phylogenetic Analysis of Evolutionary Marker Sequences

DNA sequences of 16s rRNA and D-loop region from mitochondria for each species were downloaded from the NCBI gene expression omnibus (GEO) (16s rRNA: NC007236 for *Gallus gallus*; AP003317 for WLH; NC010195 for turkey; EU755253 for duck; NC007897 for zebra finch; DQ112953 for *Homo sapiens*; NC001643 for chimpanzee; NC011120 for gorilla; and KJ567053 for macaque. D-loop: AP003322 for *Gallus gallus*; AP003317 for WLH; JF275060 for turkey; EU009397 for duck; DQ422742 for zebra finch; HQ260949 for *Homo sapiens*; NC001643 for chimpanzee; NC011120 for gorilla; and KJ567053 for macaque). Multiple 16s ribosomal DNA and D-loop sequences from five aves were aligned using Clustal Omega (version 1.2.1; [47]), respectively. The nucleotide identity of each marker was calculated from the ratio of nucleotides aligned to the corresponding sequence of the pseudo-reference (*Gallus gallus* for aves and human for primates). Pairwise genetic distances were estimated by Clustal Omega. The phylogenetic trees of the genetic distances were drawn using ClustalW2-Phylogeny v.2.1 [48].

Additionally, to compare genome-wide set of genes, we picked a group of ortholog genes where only one copy per gene exists in each genome using OMA. For 8602 sets of orthologs for aves and 13,115 sets for primates, we got nucleotide identity of each gene by pairwise sequence alignment using Clustal Omega and took a median value as a genetic distance.

### 2.4. Transcriptome Assembly

#### 2.4.1. Reference-Based Transcriptome Assembly and PRA

To map RNA-seq reads on the reference or pseudo-reference genome sequences, Tophat v.2.0.6 [49] was used with default parameters except for the maximum allowed gaps in a read (-read-gap-length) as 2 nt, manual default value to reduce wrong alignment with many indels, and the maximum allowed mismatch base number in total with variance (–N). Reads that were uniquely mapped to the reference or pseudo-reference genomes were subjected to the unique mapping rate using our custom script.

#### 2.4.2. *De Novo* Transcriptome Assembly

We used Velvet [50] and Trinity [51] for *de novo* assembly of RNA-seq reads with an optimal k-mer size of 25 nt, a minimum length of assembled contigs of 201 nt, and with the minimum k-mer coverage (-min_kmer_cov for trinity and the -cov_cutoff for velvet).

### 2.5. Virtual Chicken Genome with Random Substitution

To correlate optimal mismatch rate with genomic heterogeneity, we made synthetic genomes from genome of the *Gallus gallus* by randomly substituting nucleotides. Fifteen virtual genomes with mutation rate from 1% to 15% for each residue were generated while maintaining the transition/transversion ratio, which was observed between chicken and others species. To check the linearity of optimal mismatch rates that we found and the mutation rates, chicken RNA-seqs were mapped to the virtual chicken genomes and then the observed optimal mismatch rate for each virtual genome was plotted *versus* the mutation rate of the virtual genome, followed by regression in a linear model.

### 2.6. Quality Evaluation of Transcriptome Assembly

To evaluate the quality of transcriptome assembly, we measured the coverage (or sensitivity) of each gene, which is the fraction of the exonic region aligned by the resulting transcripts using BLAST (version 2.2.24) (Supplementary Figure S1). To build standard gene models, we collected Ensembl genes whose mature transcript was greater than 200 nt in length. To consider only expressed genes in each RNA-seq sample, isoforms of each gene with reads per million mapped reads (RPM) equal to or greater than 0.1 were chosen. All exons of the selected isoforms were unified as a standard gene model (Supplementary Figure S1). The expression level of each isoform was calculated using Cufflinks (version 2.1.1) [33].

To evaluate the overall quality of a transcriptome assembly, we next measured the reconstruction rate, the fraction of genes that have non-zero coverage to total genes, and the reconstruction rate curve, which is the cumulative fraction of genes with up to a certain coverage. The area under the cumulative curve also enables us to compare the overall performance of the transcriptome assemblers.

## 3. Results

### 3.1. Optimal Mismatch Rates for Aves PRA

A total of about 147 million (M) RNA-seq reads of four aves, including WLH (~34 M reads), turkey (~17 M reads), duck (~72 M reads), and zebra finch (~24 M reads), were respectively mapped to *Gallus gallus* genome (assembly version: Galgal4) using Tophat (version 2.0.6) [49]. To determine the optimal mapping parameter (mismatch rate) with the best unique mapping rate against a pseudo-reference, the mapping was repeated by varying the mismatch number (Supplementary Table S1).

**Figure 2:**
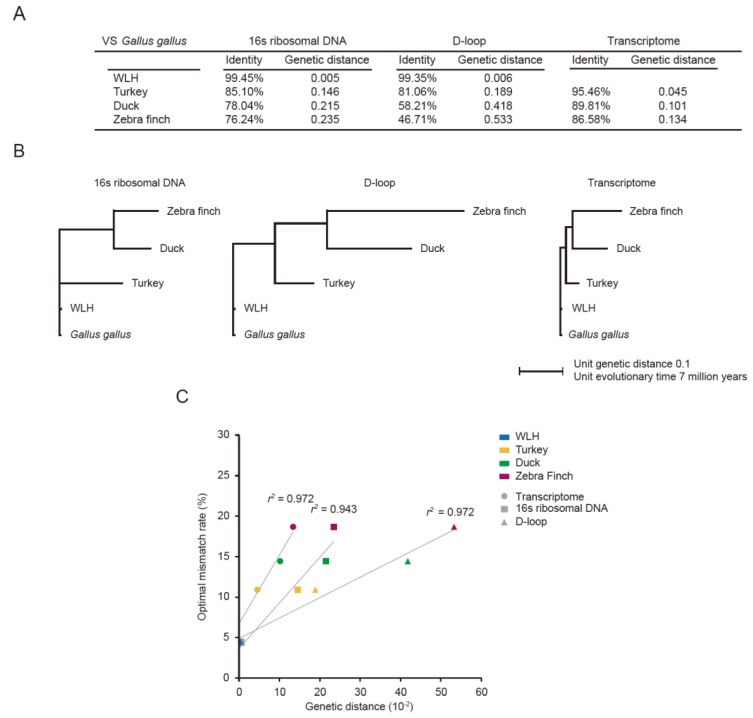
Relationship between the genetic distance of pseudo-reference species and optimal mismatch rates. (**A**) Shown are genetic distances and identities of 16s ribosomal DNAs, D-loops, and orthologous transcriptomes between four aves and Gallus gallus; (**B**) Neighbor-Joining (NJ) trees built with pairwise genetic distances. The unit genetic distance 0.1 is indicated below the trees and corresponds to approximately 7 million years ago. The pairwise genetic distance between two species was calculated by summing all heights in the tree path between the two species. The trees were constructed based on the genetic distances of 16s ribosomal DNA, D-loop and orthologous transcriptome; (**C**) The relationship between the genetic distance from the pseudo-reference (Gallus gallus) to each of the aves and the optimal mismatch rate (%) with which the unique mapping rate is maximized. The circles are drawn based on the orthologous transcriptome and the rectangles are drawn based on the 16s ribosomal DNA and the triangles are drawn based on the D-loop.

The optimal mismatch rates to achieve the best unique mapping rate were 4.44% for WLH, 10.91% for turkey, 14.44% for duck, and 18.67% for zebra finch (Supplementary Table S2A–D). The genetic distances between species were next measured using 16s ribosomal DNAs and D-loops of mitochondria and orthologous transcriptomes (Figure 2A,B). The genetic distances appeared to be linearly correlated to the optimal mismatch rates (*r*^2^ = 0.972 for orthologous transcriptome; *r*^2^ = 0.943 for 16s ribosomal DNA; *r*^2^ = 0.972 for D-loop) (Figure 2C). In addition, the evolutionary time of the D-loop is much faster than the others in the post-divergence of duck and zebra finch (Figure 2A,B).

### 3.2. Evaluation of PRAs

**Figure 3:**
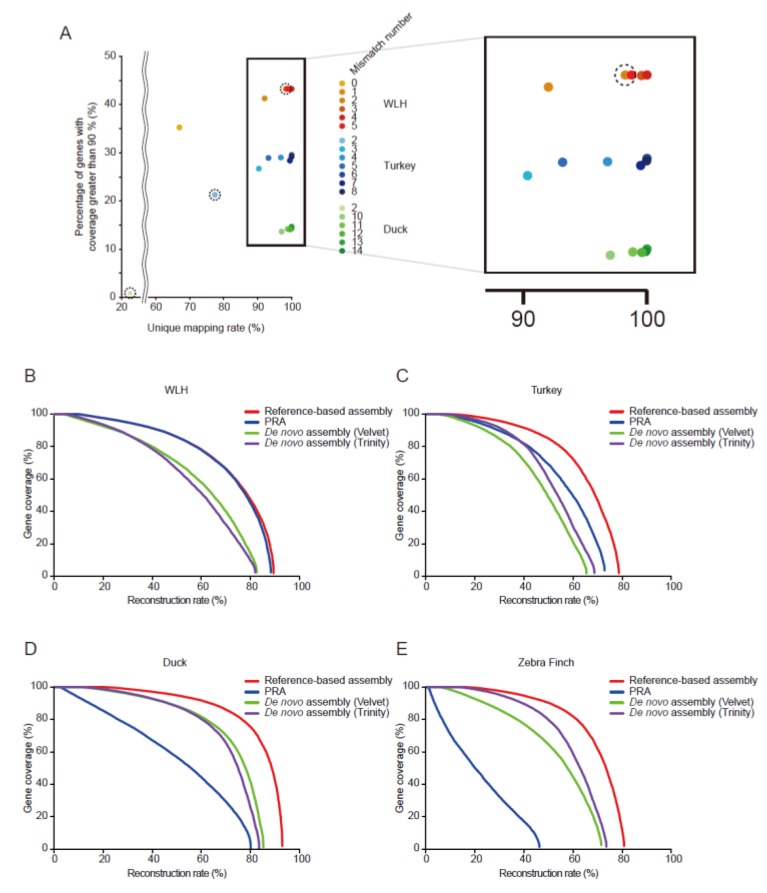
The PRAs run with the optimal mismatch rate. (**A**) The best unique mapping rate provides the transcriptome assembly with the greatest quality. Central points in dotted circles represent reconstruction rate using default parameter of Tophat, allowing only two mismatches. By varying mismatch numbers during mapping reads to a pseudo-reference (*Gallus gallus*), the unique mapping rate and the reconstruction rate for genes with greater than 90% coverage were obtained in three aves; (**B**–**E**) Gene coverages (sensitivity) of all standard genes were measured as described in the Materials and Methods and Figure S1. The genes were sorted by the gene coverage by reference-based assembly (red line), PRA (blue line), and two *de novo* assemblies (green and purple lines) in WLH (B); turkey (C); duck (D); and zebra finch (E). The reconstruction rate indicates the percentage of genes with non-zero coverage.

To confirm whether the best unique mapping rate actually conveys the PRA with the highest performance, we measured the gene coverage of the resulting transcripts against a unified gene model of isoforms with FPM equal to or greater than 0.1 (Supplementary Figure S1). Of all unified genes, the number of genes with coverage greater than 90% was maximized under the mapping condition with the greatest unique mapping rate, regardless of species (Figure 3A), suggesting that the PRAs with the best unique mapping conditions guarantee the best transcriptome assembly.

We next evaluated all resulting transcripts of the PRA under the best mapping conditions in each species in terms of the reconstruction rate and compared the results to those of the reference-based assembler, Cufflinks [33], and two *de novo* assemblers, Velvet [50] and Trinity [51] (Figure 3B–E). The PRA of WLH, a subspecies of *Gallus gallus*, was comparable to that of the reference-based assembly and outperformed those of *de novo* assemblies in terms of the reconstruction rate curve and the area under the curve (AUC) (Figure 3B). The PRA of turkey also performed better than *de novo* assembly where the genetic distance of orthologous transcriptome was 0.045 and the optimal mismatch rate was 10.91% (Figure 3C; Supplementary Table S2B). However, the reconstruction rate of PRAs for duck and zebra finch were worse than the *de novo* ones (Figure 3D,E), suggesting that the *de novo* approach is generally a better choice for remotely-related species in which the genetic distance is greater than 0.101 and the optimal mismatch rate is greater than 14.44%.

### 3.3. Optimal Mismatch Rates for Vertebrate PRAs

**Figure 4:**
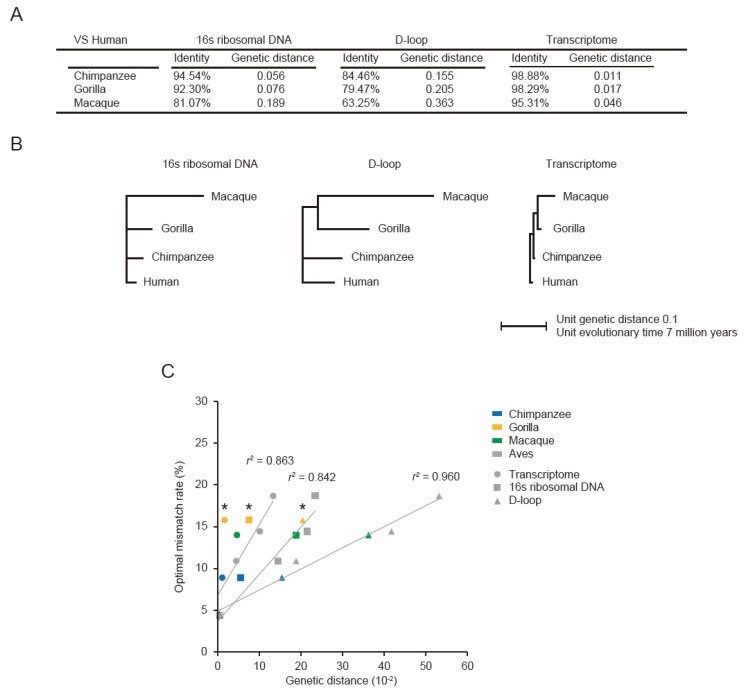

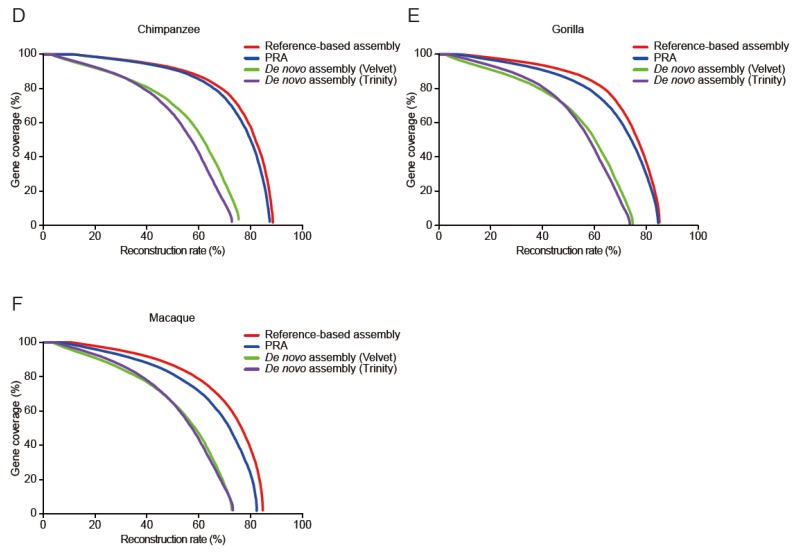
The relationship between the genetic distance of pseudo-reference species (human) and the optimal mismatch rate in primates. (**A**) Shown are genetic distances and identities of 16s ribosomal DNA, D-loop and orthologous transcriptome between three primates and the human species; (**B**) NJ trees built with pairwise genetic distances. Otherwise, as in Figure 2B; (**C**) Relationship between the genetic distance from the pseudo-reference as human to each primate and the optimal mismatch rate (%) with which the unique mapping rate is maximized; The asterisks indicate the optimal mismatch rates for gorilla, not agreed with the model. Otherwise, as in Figure 2C (**D**–**F**); Gene coverages of all gold-standard genes were measured with the reconstruction rate in chimpanzee (D); gorilla (E); and macaque (F). Otherwise, as in Figure 3B–E.

We next performed PRAs to reconstruct transcriptomes of primates including chimpanzees, gorillas, and macaques using the human genome as a pseudo-reference. To determine optimal mismatch rate of PRAs, the DNA identity and genetic distance of 16s ribosomal DNA, D-loop and orthologous transcriptome between humans and other primates were measured (Figure 4A). Similar to aves, the D-loop region evolved faster than the others, whereas the orthologous transcriptome was slower than the others (Figure 4B). To correlate the genetic distance to the optimal mismatch rate, publicly available RNA-seq reads (~58 M for chimpanzee, ~48 M for gorilla, and ~36 M for macaque) were mapped to the human genome (Supplementary Table S1). Importantly, the optimal mismatch rates given the genetic distances between humans and other primates excepting gorilla agreed with the models trained from aves (*r*^2^ = 0.863 for orthologous transcriptome; *r*^2^ = 0.842 for 16s ribosomal DNA; *r*^2^ = 0.960 for D-loop) (Figure 4C). The higher optimal mismatch rate for the RNA-seq of gorilla is most likely to be caused by the low quality of reads including many “N”.

We next evaluated the quality of primate PRAs with the optimal mismatch rate using the reconstruction rate curves and the AUC. For all chimpanzee, gorilla, and macaque, the PRAs outperformed the *de novo* assemblies and were comparable to the reference-based assembly (Figure 4D–F). Taken together, the genetic distance between humans and macaques, 0.189 for 16s ribosomal DNA, 0.363 for the D-loop region, and 0.046 for the orthologous transcriptome, were the greatest where the PRAs outperformed the *de novo* assembly.

### 3.4. Expression Level Affects the Quality of Assembled Transcripts

**Figure 5:**
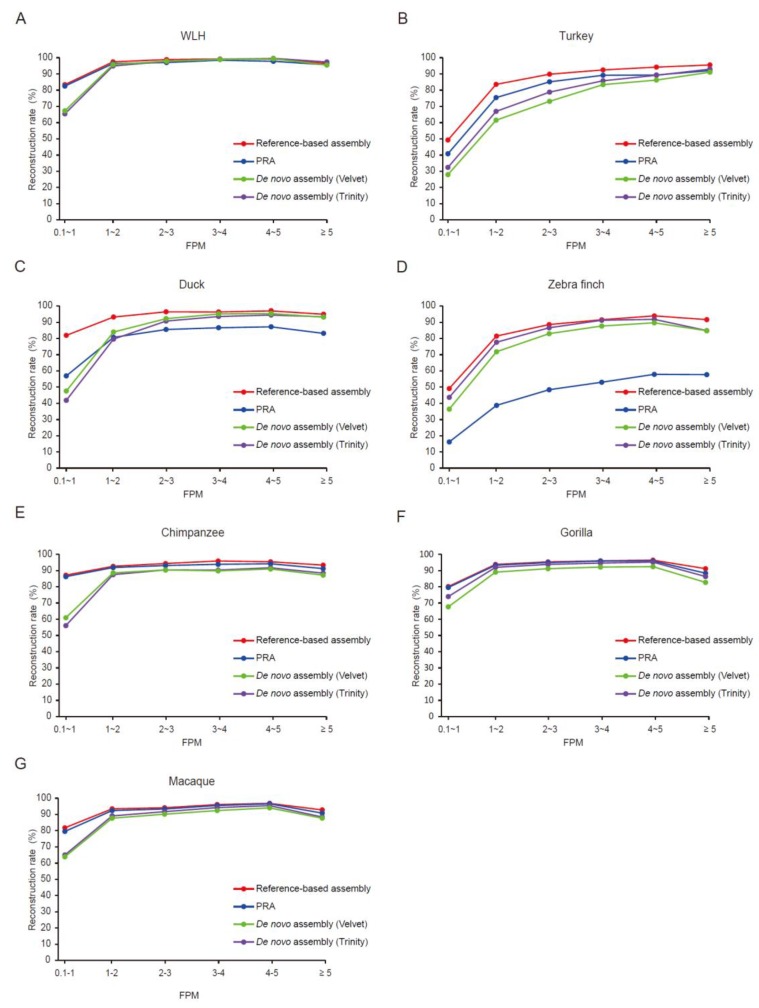
The relationship between the reconstruction rate and gene expression level. (**A**–**G**) Across genes binned by expression level (FPM), the reconstruction rates of PRA (the blue line) and other assembly approaches (reference-based assembly, red line; Velvet, green line; Trinity, purple line) were plotted in WLH (A); turkey (B); duck (C); zebra finch (D); chimpanzee (E); gorilla (F); and macaque (G).

We next sought to evaluate the PRA of each gene groups with different expression levels. PRAs performed better than did *de novo* assembly particularly in low-copy genes with fragments per million mapped reads (FPM) 0.1 to 1, as long as the genetic distance was closer than that between chicken and duck (Figure 5). Generally, the *de novo* assembly requires a sufficient read coverage to assemble short reads without a guide sequence. By contrast, the quality of the PRA was comparable to that of the *de novo* approach in medium- and high-copy genes up to the evolutionary distance between humans and macaques (Figure 5A,B,E–G) but not between chickens and ducks or between chickens and zebra finches (Figure 5C,D). Thereby, the high quality of the PRA seems to be mostly attributed to the low-copy genes.

To examine whether the PRA performs better than the *de novo* assembly in terms of overall quality of the resulting transcriptome, we calculated the AUC values of the reconstruction rate curves of the low-copy genes (with FPM 0.1 to 1) over species, ordered by genetic distance from the pseudo-reference (Supplementary Figure S2). The PRA enabled us to reconstruct transcripts with a higher or comparable sensitivity against a unified reference gene model compared to *de novo* assembly within the genetic distance of 0.101 (based on the mutation rate of orthologous transcriptomes).

### 3.5. Building an Optimal Mismatch Rate Model Using Virtual Chicken Genomes 

**Figure 6:**
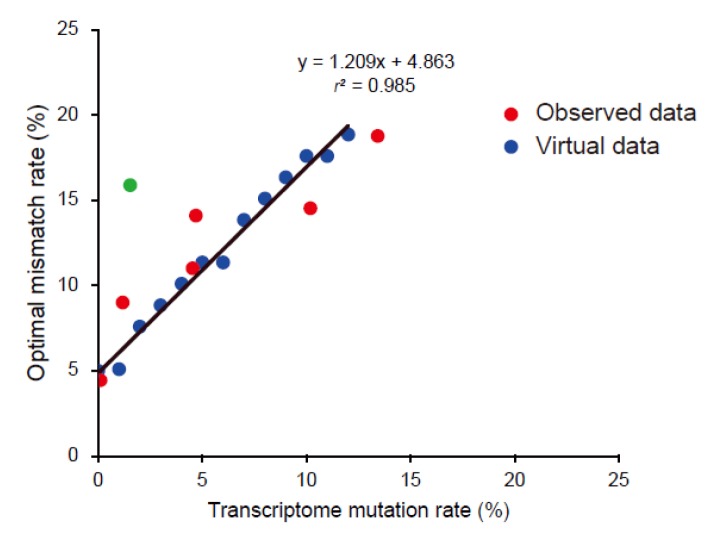
A linear regression model (black solid line) of mutation rates of virtual chicken genomes and corresponding optimal mismatch rates (blue dots). Observed data from six related species were also drawn (red dots). An outlier data (the gorilla) was indicated as a green dot.

For each virtual chicken genome with mutated sequence by a certain rate, increased by 1% up to 15%, we measured the optimal mismatch rate by mapping chicken RNA-seq data to the virtual genome. A linear regression model was built based on the virtual mutation rate and the optimal mismatch rate with a high coefficient of determination (*r*^2^ = 0.985) (Figure 6). The observed values of six species except gorilla strongly agreed with the linear model (root-mean-square error = 2.40).

### 3.6. Required Sequencing Depth for PRA

**Figure 7:**
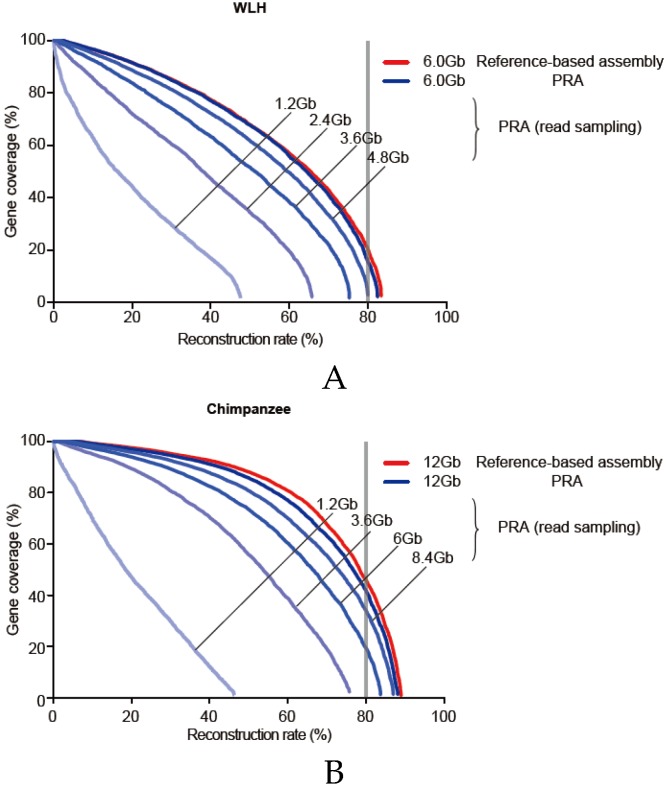
(**A**,**B**) The effect of the sequencing depth in transcriptome reconstruction. (A) The reconstruction rates of PRAs given data randomly sampled (20% (1.2 Gb), 40% (2.4 Gb), 60% (3.6 Gb), and 80% (4.8 Gb)) from the original WLH RNA-seq data were compared to those of the PRA utilizing all reads and the reference-based assembly (red line); (B) The reconstruction rates of PRAs given data randomly sampled (20% (1.2 Gb), 30% (3.6 Gb), 50% (6 Gb), and 70% (8.4 Gb)) from the original chimpanzee RNA-seq data were compared to those of the PRA utilizing all reads and the reference-based assembly (red line).

The quality of transcriptome assembly also depends on the read coverage (sequencing depth) of each gene. To examine the extent of the sequencing depth influences on PRA quality, we repeatedly performed PRAs with 20% (1.2 Gb), 40% (2.4 Gb), 60% (3.6 Gb), and 80% (4.8 Gb) reads, randomly sampled from original RNA-seq reads of WLH (6 Gb), and with 10% (1.2 Gb), 30% (3.6 Gb), 50% (6 Gb), 70% (8.4 Gb) reads, randomly sampled from original RNA-seq reads of chimpanzee (12Gb) (Figure 7). It turns out that the minimum requirements of the sequencing depth are 3.6 Gb of RNA-seq to acquire about 75% of reconstruction, and 6.0 Gb to acquire about 80% of reconstruction, similar to the quality of the reference-based assembly. Despite the genome size of chimpanzees being three times greater than that of chicken, the similar transcriptome size (50 M for chimpanzees and 42 M for chickens) results in similar minimum requirement of the sequencing depth. This provides a guideline for the RNA sequencing depth for the PRA and reference-based assembly.

## 4. Discussion

In this study, we developed a new approach for the transcriptome assembly of species for which reference genomes are not available. Our approach, PRA, takes advantage of closely related species’ genomes as a reference where RNA-seq reads will be mapped with model-based optimization of mapping conditions. The selection of genetic distance measurements can be determined based on the data availability and, if all data are available, we prefer orthologous transcriptome, 16s ribosomal DNA, and D-loop region, in turn. To date, 201 mammalian, 361 vertebrate, 2612 eukaryote, and 55,175 bacterial genomes have been sequenced and assembled into either at the scaffold or chromosome level [52]. Since at least one representative species in most genera or families include the assembled reference genome, the PRA enables us to use these genomes as pseudo-references for the other species. Although this study focused on transcriptome assembly in aves and primates, PRA is a general approach to be applied to other vertebrates. 

## 5. Conclusions

Our linear model of the optimal mismatch rate given a genetic distance between query and reference species’ genomes made us to utilize the PRA without rigorous read mappings for searching an optimal mapping parameter. The PRA better performed than the de novo transcriptome assembly is in low copy genes of closely-related species’ genomes, which lead to an extended usage of the PRA into the reference-guided genome assembly.

## Supplementary Materials

The following are available online at http://www.mdpi.com/2073-4425/7/3/10/s1. Supplementary Figure S1 and Figure S2; Table S1 and Table S2.
