# Supplementary material for: Pseudo-Reference-Based Assembly of Vertebrate Transcriptomes
Source: Genes (Basel). 2016 Feb 24;7(3):10. doi: 10.3390/genes7030010 (PMC4808791; doi:10.3390/genes7030010)
Supplement: Supplementary file 1 [file genes-07-00010-s001.docx]

**Supplementary Materials: Pseudo-Reference–Based Assembly of Vertebrate Transcriptomes**

Kyoungwoo Nam, Heesu Jeong and Jin-Wu Nam

**Table S1.** Basic statistics of RNA-seq data.

|  | **Accession Name** | **Tissue Type** | **Library Type** | **Number of Reads** | **Average Quality** | **Read Length** |
| --- | --- | --- | --- | --- | --- | --- |
| WLH | SRR1449864 | Breast muscle | Paired-end | 33671944 | 35.89 | 90 |
| Turkey | SRR478415 | Breast muscle | Paired-end | 16786786 | 36.99 | 55 |
| Duck | SRR797835 | Liver | Paired-end | 72023410 | 35.77 | 90 |
| Zebra finch | SRR1198326 | Brain | Single-end | 24331046 | 33.12 | 75 |
| Chimpanzee | SRR1758917 | Brain | Paired-end | 58485618 | 33.87 | 101 |
| Gorilla | SRR649365 | Brain | Single-end | 47869584 | 26.13 | 76 |
| Macaque | SRR1602563 | Brain | Paired-end | 35808821 | 34.53 | 100 |

**Table S2.** Unique mapping rates (%) given mismatch rates to the corresponding pseudo-reference genomes in WLH (A), turkey (B), duck (C), zebra finch (D), chimpanzee (E), gorilla (F), and macaque (G).

A. WLH

| **Mismatch Rate (Mismatch / Read Length)** | **Unique Mapping Rate** |
| --- | --- |
| 0.00% (0/90) | 53.99% |
| 1.11% (1/90) | 74.18% |
| 2.22% (2/90) | 79.19% |
| 3.33% (3/90) | 80.41% |
| 4.44% (4/90) | 80.59% |
| 5.56% (5/90) | 80.24% |
| 8.89% (8/90) | 78.38% |

B. Turkey

| **Mismatch Rate (Mismatch / Read Length)** | **Unique Mapping Rate** |
| --- | --- |
| 5.45% (3/55) | 48.86% |
| 7.27% (4/55) | 52.35% |
| 9.09% (5/55) | 50.40% |
| 10.91% (6/55) | 54.08% |
| 12.73% (7/55) | 54.07% |
| 14.54% (8/55) | 53.81% |
| 21.82% (12/55) | 52.15% |

C. Duck

| **Mismatch Rate (Mismatch / Read Length)** | **Unique Mapping Rate** |
| --- | --- |
| 11.11% (10/90) | 25.56% |
| 12.22% (11/90) | 26.04% |
| 13.33% (12/90) | 26.26% |
| 14.44% (13/90) | 26.34% |
| 15.56% (14/190) | 26.32% |
| 16.67% (15/90) | 26.23% |
| 28.89% (26/90) | 24.29% |

D. Zebra finch

| **Mismatch Rate (Mismatch / Read Length)** | **Unique Mapping Rate** |
| --- | --- |
| 14.67% (11/75) | 19.55% |
| 16.00% (12/75) | 19.68% |
| 17.33% (13/75) | 19.77% |
| 18.67% (14/75) | 19.78% |
| 20.00% (15/75) | 19.75% |
| 21.33% (16/75) | 19.71% |
| 37.33% (28/75) | 19.30% |

E. Chimpanzee

| **Mismatch Rate (Mismatch / Read Length)** | **Unique Mapping Rate** |
| --- | --- |
| 4.95% (5/101) | 83.78% |
| 5.94% (6/101) | 84.65% |
| 6.93% (7/101) | 85.09% |
| 7.92% (8/101) | 95.25% |
| 8.91% (9/101) | 95.29% |
| 9.90% (10/101) | 85.24% |
| 17.82% (18/101) | 83.82% |

F. Gorilla

| **Mismatch Rate (Mismatch / Read Length)** | **Unique Mapping Rate** |
| --- | --- |
| 10.53% (8/76) | 69.42% |
| 11.84% (9/76) | 69.81% |
| 13.16% (10/76) | 69.98% |
| 14.47% (11/76) | 70.085% |
| 15.79% (12/76) | 70.087% |
| 17.10% (13/76) | 70.06% |
| 31.58% (24/76) | 67.15% |

G. Macaque

| **Mismatch Rate (Mismatch / Read Length)** | **Unique Mapping Rate** |
| --- | --- |
| 12.00% (12/100) | 67.47% |
| 13.00% (13/100) | 67.63% |
| 14.00% (14/100) | 67.68% |
| 15.00% (15/100) | 67.65% |
| 16.00% (16/100) | 67.58% |
| 17.00% (17/100) | 67.46% |
| 28.00% (28/100) | 66.15% |


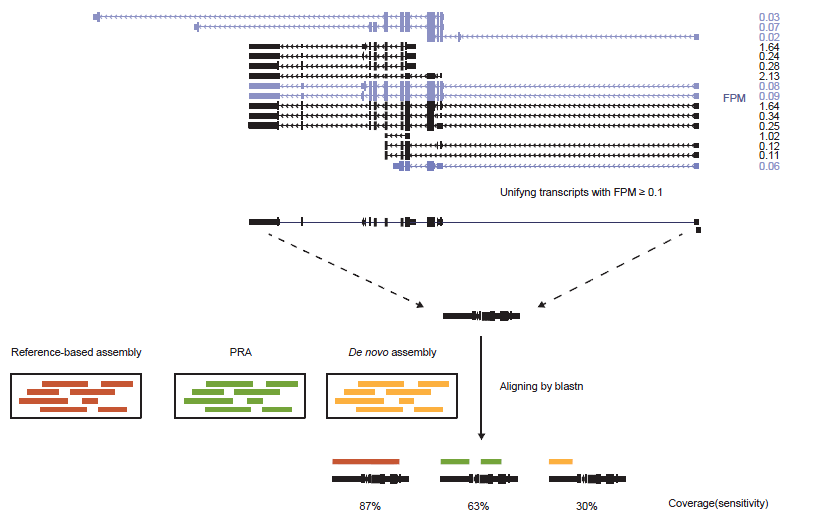


**Figure S1.** A schematic flow to measure the gene coverage (sensitivity) of each resulting assembly. Of all transcript isoforms of a gene, exons of all isoforms with FPM ≥ 0.1 were unified as a standard gene model. The gene coverage was measured with the nucleotide fraction of a certain gene covered by the resulting assembly of each approach (reference-based assembly, orange bars; PRA, green bars; de novo assembly, yellow bars).


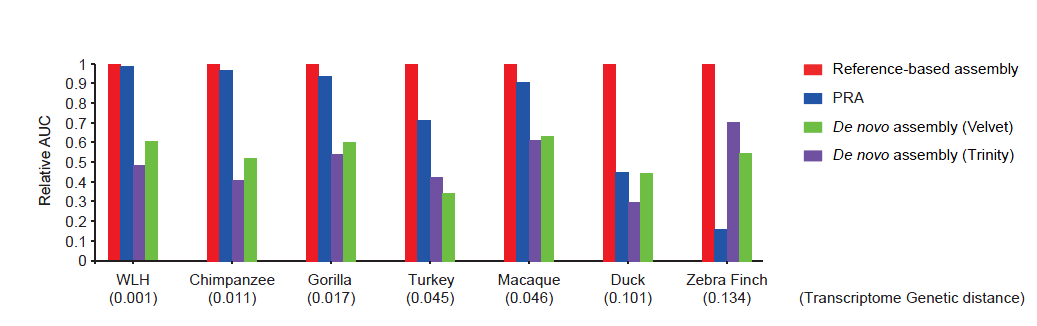


**Figure S2.** Comparison of transcriptome assembly approaches in terms of overall reconstruction rate. The overall reconstruction rates were estimated as the AUCs relative to those of the reference-based assemblies across seven query species. The species were sorted by the genetic distance based on the mutation rate of orthologous transcriptomes from the corresponding pseudo-reference
